# Validity and reliability of the osteoporotic fracture treatment score (OF score) and outcomes across various treatments in osteoporosis vertebral compression fracture patients

**DOI:** 10.1186/s13018-024-05244-3

**Published:** 2024-11-13

**Authors:** Korawish Mekariya, Borriwat Santipas, Harit Khamnurak, Wilasinee Sirichativapee, Ekkapoj Korwutthikulrangsri, Monchai Ruangchainikom, Werasak Sutipornpalangkul

**Affiliations:** 1 Department of Orthopaedic Surgery, Faculty of Medicine Siriraj Hospital, Mahidol University, Bangkok, Thailand; 2 Department of Orthopaedic Surgery, Faculty of Medicine, Khon Kaen University, Khon Kaen, Thailand

**Keywords:** Osteoporotic vertebral compression fractures, OF score, OF-classification, German Society for Orthopaedic and Trauma Surgery (DGOU), Validity and reliability

## Abstract

**Background:**

Osteoporotic vertebral compression fractures (OVF) are prevalent and substantially impact healthcare systems and patients’ quality of life. The osteoporotic fracture treatment score (OF score), developed by the German Society of Orthopedics and Trauma (DGOU), guides surgical decisions, but its reliability and validity are underexplored. This study assessed the OF score’s inter- and intraobserver reliability, validated its treatment recommendations, and investigated intermediate outcomes of different DGOU-recommended surgical strategies for OVF.

**Methods:**

A retrospective cohort study was conducted. Inter- and intraobserver reliability of the OF score and its subcomponents were analyzed using clinical and radiographic data. Validity was assessed by comparing the OF score’s recommended treatments with actual treatments received. Outcomes at the final follow-up were back pain visual analog scale, Oswestry Disability Index, EQ-VAS, EQ-5D-5 L, adjacent fracture incidence, local kyphotic angle, and reoperation rates. Patients with at least 1-year follow-up were included.

**Results:**

A total of 157 patients (84.7% female; mean age 74.2 ± 10.5 years) were evaluated. The most frequent osteoporotic fracture (OF) types were OF4 (49.0%) and OF3 (40.8%). The OF score demonstrated good interobserver reliability (ICC = 0.77, 95% CI: 0.65–0.86) and intraobserver reliability (ICC = 0.83, 95% CI: 0.72–0.90). Kappa values for subcomponents ranged from 0.57 to 0.89. Excluding patients with indeterminate recommendations (OF score = 6), 82.9% received treatments concordant with OF score recommendations. Receiver operating characteristic curve analysis showed an area under the curve of 0.77 (95% CI: 0.67–0.86); an OF score cutoff > 6.5 predicted actual treatment with 87.9% sensitivity and 61.0% specificity. All surgical treatments showed comparable improvements in clinical outcomes. However, patients treated with stand-alone cement augmentation (CA) had less local kyphotic angle correction (*P* = 0.004) and greater postoperative kyphotic progression (*P* < 0.001) than those undergoing short-segment (SS-PI) or long-segment instrumentation (LS-PI). No significant differences in adjacent fractures or complications were observed.

**Conclusions:**

The OF score is a reliable and valid system with good discriminative ability for surgical decision-making in OVF patients. CA, SS-PI, and LS-PI are viable options with comparable functional outcomes. However, in OF3 or OF4 fractures, caution is advised due to lesser kyphosis correction and greater kyphotic progression with CA compared to SS-PI or LS-PI, as recommended by the DGOU.

## Background

Osteoporotic vertebral compression fractures (OVF) are fragility fractures of the vertebral body that primarily occur in the thoracolumbar spine [1]. These fractures are the most prevalent osteoporotic fractures, with a global incidence of one every 22 s among individuals aged 50 and older, regardless of sex [2, 3]. OVF increase morbidity and mortality [4, 5] and impose substantial healthcare costs, estimated at up to $1 billion annually [6, 7]. Also, the incidence of OVF and surgical intervention rates are rising due to population aging [8, 9].

Despite its prevalence, consensus on OVF management remains elusive. Treatment is complex and often controversial, particularly regarding surgical intervention thresholds and appropriate surgical methods [10, 11]. Recently, the “Osteoporotic Fractures” working group within the Spine Section of the German Society of Orthopedics and Trauma (DGOU) developed the osteoporotic fracture (OF) classification and osteoporotic fracture treatment score (OF score) [12, 13], which have been widely adopted. The OF classification includes five types: OF1 (no vertebral deformation), OF2 (deformation with minimal posterior wall involvement), OF3 (deformation with significant posterior wall involvement), OF4 (vertebral frame integrity loss, vertebral body collapse, or pincer-type fracture), and OF5 (distraction or rotational injury). The OF score assesses seven components to guide treatment decisions: the OF classification, bone mineral density, ongoing fracture process, pain, neurological status, mobility status, and patient health status. The scores categorize the recommendations as “nonsurgical” (0–5 points), “nonsurgical or surgical” (6 points), or “surgical” (> 6 points). However, the reliability, validity, and outcomes of the OF classification and OF score have been evaluated in few studies. This study aimed to assess the reliability and validity of the OF score and its treatment recommendations. Additionally, we examined the intermediate outcomes of various surgical strategies for OVF patients.

## Methods

### Study design and ethical approval

This retrospective cohort study was conducted at a single-center tertiary care hospital. The Siriraj Institutional Review Board of the Faculty of Medicine of Siriraj Hospital, Mahidol University, Bangkok, Thailand, approved the study protocol (COA no. si 864/2022). The requirement for written informed consent was waived because of the retrospective and anonymous nature of the study.

## Study population

Data were sourced from the institution’s spinal registry. This retrospective analysis included patients treated for thoracolumbar OVF from 2016 to 2021.

## Inclusion and exclusion criteria

The inclusion criteria were the following:

- Postmenopausal women and men aged ≥ 50 years who had been diagnosed with thoracolumbar OVF presenting with focal back pain at the fracture level for 4 weeks or less.
- The presence of pain or local tenderness consistent with imaging findings, including X-ray, computerized tomography (CT), or magnetic resonance imaging (MRI).
- The availability of clinical and radiological data for preoperative OF score assessment.

The exclusion criteria were as follows:

- The presence of thoracolumbar fractures that were secondary to spinal tumor diseases (e.g., primary spinal tumors such as spinal hemangioma, spinal metastasis, and hematologic malignancies like multiple myeloma).
- Spinal infections (e.g., spondylodiscitis).
- Patients with previous spinal surgery.

## Data collection

Baseline demographic data, including age, sex, body mass index, history of trauma, underlying diseases, American Society of Anesthesiologists physical status classification, and medication usage, were retrieved. Clinical variables related to pain, neurological status, patient mobility, and bone mineral density were also collected.

## OF score assessment

Three orthopedic surgeons (K.M., B.S., and H.K.), blinded to patient details and treatments, independently reviewed the images (X-ray, CT, or MRI) to classify patients via the OF classification and calculate the OF score on the basis of the clinical and radiographic data. The total OF score was further categorized into “stable,” “potentially unstable,” or “unstable,” according to the guidelines for recommended treatments.

Before evaluating the OF score, all reviewers reviewed the OF classification and OF scoring systems described in the original articles by Schnake et al. [13] and Blattert et al. [12]. Discrepancies in OF classifications and OF scores were resolved by a fourth reviewer (W.S.) through discussion, establishing a consensus classification and score for validity analysis. Interobserver agreement was assessed by comparing the initial classifications of the three evaluators. Intraobserver reliability was evaluated 6 weeks later by the same raters, using patient cases presented in random order to minimize recall bias. To assess the validity of the OF score in predicting treatment, the recommended treatments based on the consensus OF score were compared with the treatments administered to each patient.

### Outcome measures

Patients with at least 1 year of follow-up were included in the outcome analysis. The primary outcomes measured were back pain via the visual analog scale (VAS; 0–100 mm, where 0 indicates “no pain” and 100 represents “the worst pain”), the Oswestry Disability Index (ODI), the EQ-5D-5 L, and the EQ-VAS. These parameters were assessed at baseline and at the 1-year follow-up.

**Fig. 1:**
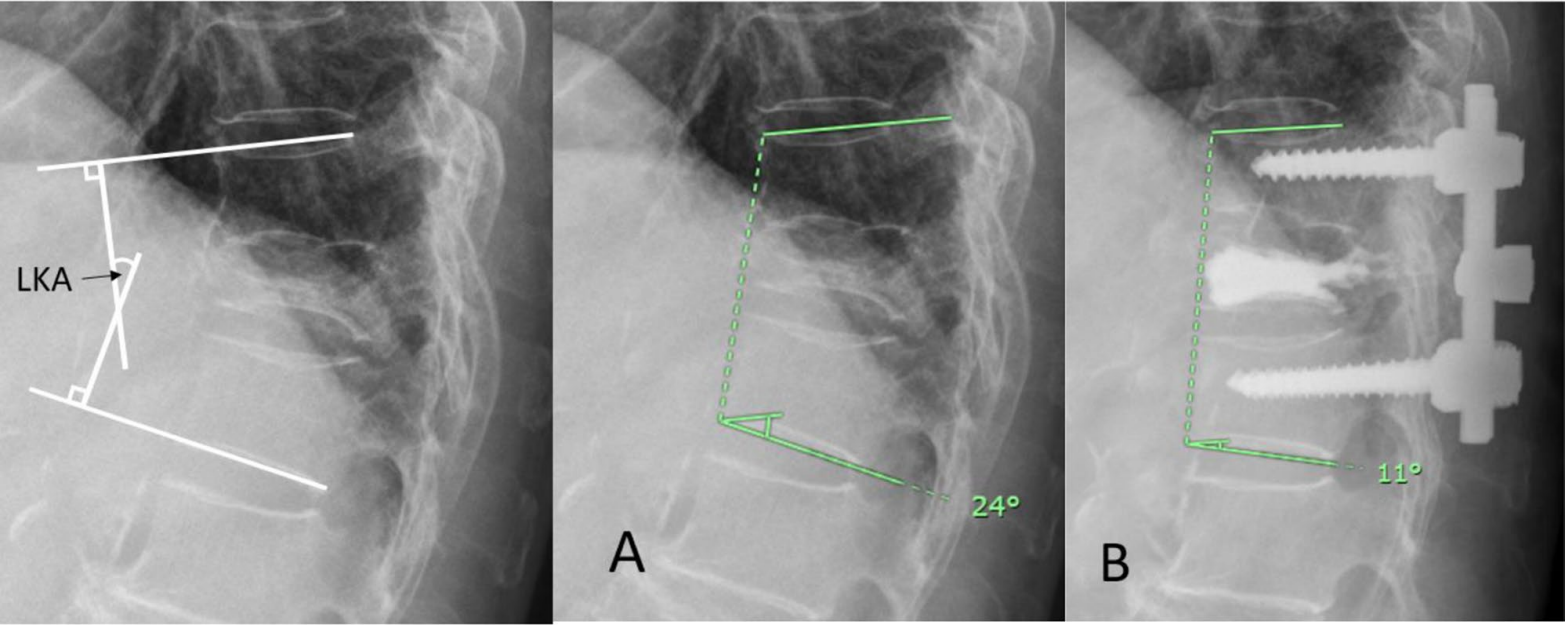
Measurement of local kyphotic angles (LKAs). The angles are measured at the superior and inferior endplates of vertebrae adjacent to the fracture. The kyphotic correction was determined by comparing the preoperative and postoperative LKAs. **(A)** A preoperative LKA of 24 degrees. **(B)** A kyphotic correction of 13 degrees

Radiographic outcomes were evaluated using lateral plain radiographs in the upright position. Sagittal Cobb angles were measured at the superior and inferior endplates of the vertebra adjacent to or spanning the fracture site, defined as the local kyphotic angle (Fig. 1). Kyphotic correction was determined by comparing the preoperative and postoperative local kyphotic angles. Postoperative kyphotic progression was defined as an increase in the local kyphotic angle of more than 10 degrees compared with the immediate postoperative measurement. Additionally, the rates of adjacent-level fractures and reoperations were recorded and analyzed.

## Surgical treatment classification

Surgical treatments for OVF were classified according to the DGOU treatment recommendations [12]:

- **Stand-alone cement augmentation (CA)**: This technique includes vertebroplasty or kyphoplasty without posterior instrumentation.
- **Short-segment posterior instrumentation (SS-PI)**: This method involves instrumentation of one segment above and below the fractured vertebra.
- **Long-segment posterior instrumentation (LS-PI)**: This technique requires instrumentation of at least two segments above and below the fractured vertebra.

## Sample size calculation

Sample size calculations were performed to ensure adequate power for assessing interobserver reliability and the validity of the OF score. Using Walter’s formula with a minimum acceptable intraclass correlation coefficient (ICC) of 0.6, an expected ICC of 0.75, a significance level of α = 0.05, a power of 80%, and three raters, a total of 67 patients were needed. For the validity analysis, the sample size was estimated via a proportion formula. Assuming a proportion (p) of 71% based on Ullrich et al. [14], an error margin (d) of 10%, and α = 0.05, a total of 157 samples were necessary.

### Statistical analysis

Interobserver and intraobserver reliability for total OF scores among the three raters were assessed using a two-way mixed-effects model and the ICC. Each OF score component—the OF classification, bone mineral density, ongoing fracture, pain, neurological deficit, mobilization, and health status—was evaluated for interobserver agreement via Fleiss’s kappa and for intraobserver agreement using Cohen’s weighted kappa. Agreement levels were interpreted on the basis of Koo and Li’s grading [15] for ICC values and Landis and Koch’s grading [16] for kappa values.

The validity of treatment decisions based on the OF score was determined by calculating the percentage of correct treatment predictions, sensitivity, specificity, positive predictive value, and negative predictive value. Receiver operating characteristic (ROC) curve analysis with Youden’s index was performed to estimate the area under the curve (AUC) and identify the OF score cutoff value, along with its sensitivity and specificity, for surgical treatment recommendations.

Continuous data are presented as means ± standard deviations for normally distributed variables and as medians with interquartile ranges for nonnormally distributed variables. Categorical data are described as numbers and percentages. Comparisons of continuous data were performed via Student’s t test or the Mann–Whitney U test, whereas categorical data were compared using the chi-square test or Fisher’s exact test. A *P* value of less than 0.05 was considered statistically significant. Bonferroni correction was applied to adjust for multiple comparisons, setting the significance threshold at *P* < 0.013 (0.05 divided by 3). All the statistical analyses were conducted with IBM SPSS Statistics, version 29.0.2.0 (IBM Corp, Armonk, NY, USA).

## Results

### Study population

A total of 181 patients were initially eligible for the study. After applying the exclusion criteria; 12 fractures secondary to spinal tumors, 8 fractures secondary to spinal infections, and 4 fractures with previous spinal surgery, 157 patients were enrolled in this study. Of these, 132 patients had at least one year of follow-up and were included in the intermediate outcome analysis.

### Baseline demographic and clinical characteristics

The baseline demographic and clinical characteristics are summarized in Table 1. The mean age of the participants was 74.2 ± 10.5 years, with 84.7% being female. A history of trauma was reported by 54.8% of the patients. Most fractures occurred at the thoracolumbar junction (T11–L2; 83.4% of cases). According to the OF classification, OF4 was the most prevalent type (49.04%, *n* = 77), followed by OF3 (40.8%, *n* = 64), OF2 (8.9%, *n* = 14), and OF5 (1.3%, *n* = 2).

**Table 1:** Demographic and clinical characteristics of the study population

Characteristics (*n* = 157)	*N* (%) | Mean ± SD
Age (yrs)	74.2 ± 10.5
Female	133 (84.7%)
BMI (kg/m^2^)	23.7 ± 3.6
Reported history of trauma	86 (54.8%)
Currently on an anti-osteoporotic drug	18 (11.5%)
Risk factors	
• DM	41 (26.1%)
• Corticosteroids	8 (5.1%)
• Smoking	4 (2.5%)
• CKD	15 (9.6%)
ASA classification	
• Class 1	6 (3.8%)
• Class 2	136 (86.6%)
• Class 3	15 (9.6%)
Location of fracture	
• Mid thoracic (T3-T10)	5 (3.2%)
• Thoracolumbar junction (T11-L2)	131 (83.4%)
• Lumbar (L2-L5)	21 (13.4%)
Presence of neurological deficits	25 (15.9%)

Abbreviations ASA, American Society of Anesthesiologists (physical status classification); BMI, body mass index; CKD, chronic kidney disease; DM, diabetes mellitus

### Reliability of the OF score

The interobserver reliability for the total OF score among the three raters was excellent, with an ICC of 0.774 (95% CI: 0.653–0.858), indicating good agreement. Intraobserver reliability demonstrated an ICC of 0.833 (95% CI: 0.721–0.900), also reflecting good agreement. Detailed analyses of each OF score component’s interobserver and intraobserver reliability and their levels of agreement are presented in Table 2.

**Table 2:** Interobserver and intraobserver reliability of OF-score components

	Interobserver reliability			Intraobserver reliability		
**OF score component**	Kappa ^a^	95% CI	Agreement	Kappa ^a^	95% CI	Agreement
OF classification	0.608	0.498–0.719	Substantial	0.646	0.438–0.854	Substantial
BMD	0.894	0.748-1.000	Near perfect	0.961	0.885-1.000	Near perfect
Ongoing fracture	0.572	0.426–0.718	Moderate	0.686	0.490–0.882	Substantial
Pain VAS	0.872	0.726-1.00	Near perfect	0.923	0.819-1.00	Near perfect
Neurological deficit	0.843	0.698–0.989	Near perfect	0.846	0.701–0.991	Near perfect
Mobilization	0.667	0.520–0.813	Substantial	0.767	0.604–0.929	Substantial
Health status	0.701	0.569–0.833	Substantial	0.860	0.726–0.994	Substantial

^a^ Cohen’s weighted kappa was estimated using linear weights

Abbreviations BMD, bone mineral density; OF, osteoporotic fracture; VAS, visual analog scale

### Validity of the OF score for treatment prediction

Among the 157 patients (Table 3), 116 (73.89%) underwent surgical treatment, whereas 41 (26.11%) received conservative treatment. On the basis of the OF score, 28 patients (17.83%) were recommended for conservative treatment (OF score < 6), and 118 patients (75.16%) were recommended for surgical treatment (OF score ≥ 7). Eleven patients (7%) had indeterminate recommendations (OF score = 6); of these, 6 were treated conservatively, and 5 underwent surgery. Overall, 121 patients (82.88%) received treatment consistent with the OF score recommendations.

**Table 3:** Comparison between OF-score recommended treatment and actual treatment

	OF-score recommendation			
**Actual treatment**	Conservative	Indeterminate	Surgical	Total
Conservative	19	6	16	41
Surgical	9	5	102	116
Total	28	11	118	

Abbreviations OF, osteoporotic fracture

**Fig. 2:**
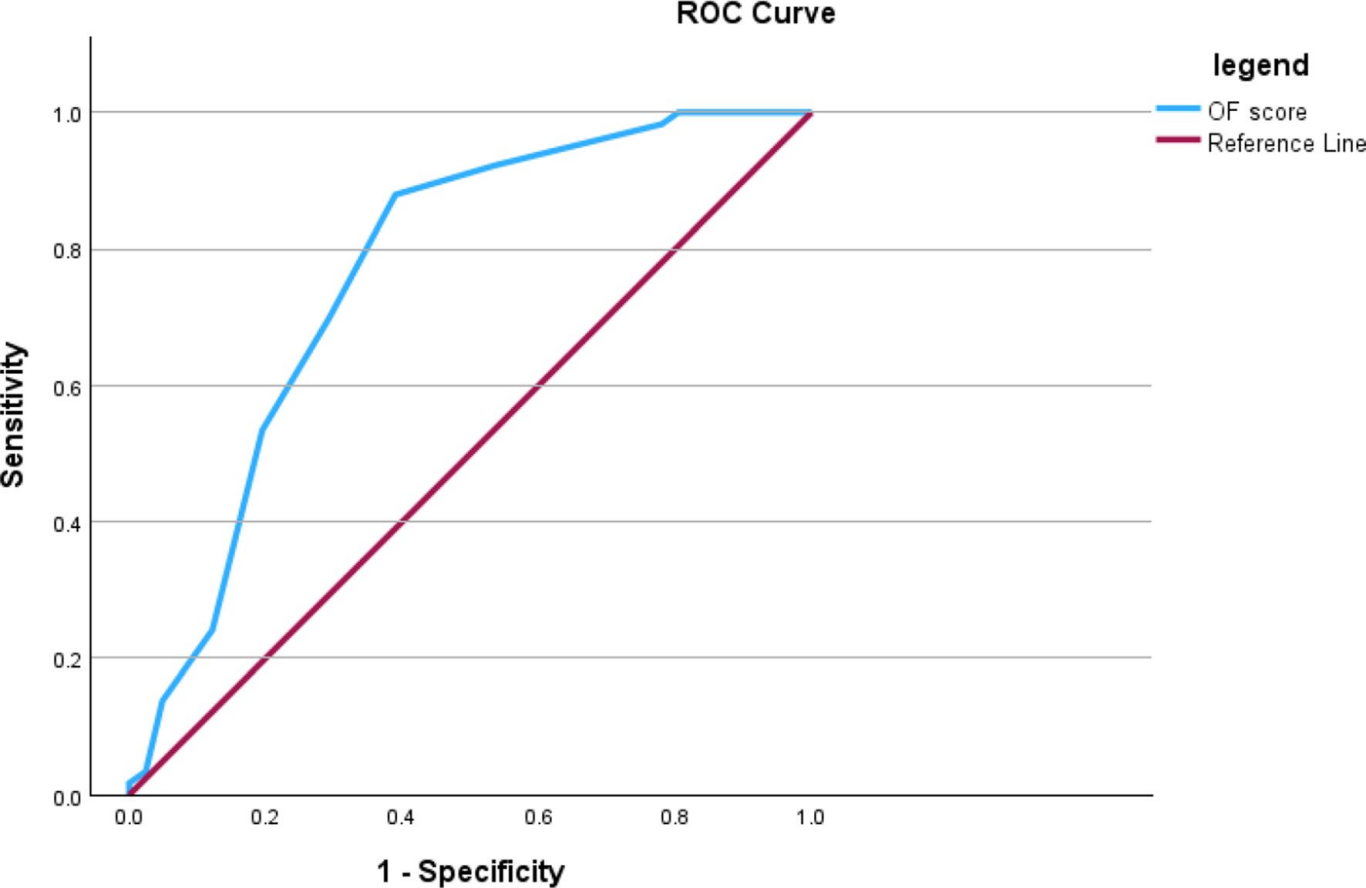
Receiver operating characteristic curve of OF score for predicting surgical versus conservative treatment. The cutoff value of 6.5 demonstrates a sensitivity of 87.9% and a specificity of 61%, with an area under the curve (AUC) of 0.766 (95% CI: 0.670–0.861)

The validity of the OF score in predicting surgical treatment is summarized in Table 4. The ROC curve analysis revealed an AUC of 0.77 (95% CI: 0.67–0.86), indicating fair discriminative ability [17]. When a cutoff OF score of > 6.5 was used, the sensitivity and specificity for predicting surgical treatment were 87.9% and 61.0%, respectively **(**Fig. 2**)**.

**Table 4:** Summary of OF-score validity for treatment predictions

	% Correctness	Sensitivity	Specificity	PPV	NPV
OF-score proposed treatment vs. actual treatment	82.88%	91.89%	54.29%	86.44%	67.86%

Patients with an indeterminate OF score of 6 were excluded from the validity calculations

Abbreviations NPV, negative predictive value; OF score, osteoporotic fracture treatment score; PPV, positive predictive value

### Clinical outcomes of surgical treatments

Among the 132 patients included in the outcome analysis, 106 (80.3%) received surgical treatment, while 26 (19.7%) were treated nonsurgically. The surgically treated patients were categorized into three groups: CA (*n* = 49), SS-PI (*n* = 29), and LS-PI (*n* = 28). The demographic and clinical characteristics of these groups are detailed in Table 5. There were no significant differences in demographic characteristics or clinical risk factors across the groups. The majority of patients in each group were classified as OF4, followed by OF3, and most fractures were located at the thoracolumbar junction. Notably, the LS-PI group had a significantly greater mean OF score (9.1 ± 1.9; *P* = 0.011) than did the CA group. Additionally, neurological deficits were more prevalent in the LS-PI group (*P* < 0.001). The median follow-up duration was consistent across the groups at 48 weeks.

**Table 5:** Baseline demographic and clinical characteristics by treatment group

*N* (%) | Mean ± SD | Median (Q1, Q3)	CA (*N* = 49)	SS-PI (*N* = 29)	LS-PI (*N* = 28)	*P* value
Female	43 (87.8%)	22 (75.9%)	24 (85.7%)	0.368
Age at surgery (yrs)	74.8 ± 8.3	72.3 ± 8.9	71.9 ± 7.8	0.268
BMI	23.8 ± 3.8	23.6 ± 3.9	23.5 ± 2.9	0.950
Risk factors				
• DM	8 (16.3%)	7 (24.1%)	9 (32.1%)	0.273
• Corticosteroids	5 (10.2%)	1 (3.4%)	0 (0%)	0.147
• Smoking	2 (4.1%)	1 (3.4%)	1 (3.6%)	0.988
• CKD	4 (8.2%)	4 (13.8%)	3 (10.7%)	0.731
• ASA classification ≥ 3	5 (10.2%)	6 (20.7%)	1 (3.6%)	0.130
OF classification				0.118
• OF-2	4 (8.2%)	2 (6.9%)	0 (0%)	
• OF-3	22 (44.9%)	11 (37.9%)	6 (21.4%)	
• OF-4	23 (46.9%)	16 (55.2%)	21 (75.0%)	
• OF-5	0 (0%)	0 (0%)	1 (3.6%)	
OF score	7.9 ± 1.6	8.7 ± 1.7	9.1 ± 1.9	0.011*
Location of fracture				0.054
• Mid thoracic (T3-T10)	2 (4.1%)	0 (0.0%)	1 (3.6%)	
• Thoracolumbar junction (T11-L2)	42 (85.7%)	22 (75.9%)	27 (96.4%)	
• Lumbar (L2-L5)	5 (10.2%)	7 (24.1%)	0 (0%)	
Presence of neurological deficits	1 (2.0%)	8 (27.6%)	12 (42.9%)	< 0.001*
Follow-up	48.0 (48.0, 48.0)	48.0 (48.0, 48.0)	48.0 (48.0, 54.0)	0.055

* The *P* value was calculated via one-way ANOVA

Abbreviations ASA, American Society of Anesthesiologists (physical status classification); BMI, body mass index; CA, cement augmentation; CKD, chronic kidney disease; DM, diabetes mellitus; LS-PI, long-segment posterior instrumentation; OF, osteoporotic fracture; OF score, osteoporotic fracture treatment score; SS-PI, short-segment posterior instrumentation

Preoperative measures (back pain VAS score, ODI, EQ-VAS score, EQ-5D-5 L score, and initial local kyphotic angle) were not significantly different between the treatment groups (*P* > 0.05; Table 6). Postoperatively, kyphotic reduction varied significantly among the groups, with the LS-PI group achieving the most substantial reduction (median 13.0°, *P* = 0.004). Pairwise comparisons revealed significant differences between the CA and SS-PI groups and between the CA and LS-PI groups (*P* = 0.002). Kyphotic progression also differed significantly, with the CA group experiencing greater progression (mean 15.3°) than the SS-PI (9.4°) and LS-PI (5.1°) groups did (*P* < 0.001). Pairwise comparisons revealed significant differences in kyphotic progression between CA and SS-PI as well as between CA and LS-PI (*P* < 0.001).

**Table 6:** Preoperative clinical outcomes, radiographic outcomes, and complications by treatment group

*N* (%) | Mean ± SD | Median (Q1, Q3)	CA (*N* = 49)	SS-PI (*N* = 29)	LS-PI (*N* = 28)	*P* value
Back pain VAS preop	63.9 ± 27.3	60.8 ± 25.4	53.6 ± 30.4	0.347
ODI preop	60.0 (46.0, 75.0)	63.3 (46.11, 77.6)	66.7 (45.8, 75.2)	0.701
EQ-VAS preop	50.0 (40.0, 70.0)	50.0 (25.0, 70.0)	50.0 (30.0, 55.0)	0.457
EQ-5D-5 L preop	0.4 ± 0.4	0.3 ± 0.3	0.4 ± 0.4	0.840
LKA preop	19.1 ± 17.3	22.1 ± 12.1	23.3 ± 13.2	0.624
Kyphosis reduction	3.0 (0.0, 8.0)	10.5 (5.8, 15.0)	13.0 (6.3, 28.3)	0.004*
Kyphosis progression	15.3 ± 6.8	9.4 ± 8.6	5.1 ± 7.1	< 0.001*
Complications	11 (22.4%)	5 (17.2%)	2 (7.1%)	0.227
Adjacent fracture	9 (20.5%)	6 (23.1%)	6 (22.2%)	0.964
Re-operation	3 (6.3%)	4 (14.3%)	1 (3.8%)	0.309

*P-value has statistically significance (*P* < 0.05)

Abbreviations CA, cement augmentation; LKA, local kyphotic angle; LS-PI, long-segment posterior instrumentation; ODI, Oswestry Disability Index; OF, osteoporotic fracture; SS-PI, short-segment posterior instrumentation; VAS, visual analog scale

The complication and reoperation rates did not differ significantly between the groups (*P* = 0.227 and *P* = 0.309, respectively). Adjacent-level fractures occurred in 20.5% of CA patients, 23.1% of SS-PI patients, and 22.2% of LS-PI patients, with no significant differences (*P* = 0.964) **(**Table 6). Regardless of treatment type, all patients showed significant improvements in back pain VAS, ODI, EQ-5D-5 L, and EQ-VAS scores, with no significant differences between the treatment groups (Table 7).

**Table 7:** Mean and median change in clinical and health-related quality of life outcomes by treatment group

Mean change Mean ± SD | Median (Q1, Q3)	CA (*N* = 49)	SS-PI (*N* = 29)	LS-PI (*N* = 28)	*P* value
Back pain VAS	-41.8 ± 39.1	-39.1 ± 17.7	-35.0 ± 26.6	0.724
ODI	-14.0 (-28.9, -5.8)	-18.0 (-23.3, -9.4)	-22.0 (-34.0, -8.0)	0.423
EQ-VAS	20.0 (0.0, 30.0)	20.0 (6.0, 30.0)	20.0 (8.8, 40.0)	0.952
EQ-5D-5 L	0.2 (0.1, 0.4)	0.3 (0.0, 0.5)	0.1 (0.1, 0.6)	0.930

Abbreviations CA, cement augmentation; LS-PI, long-segment posterior instrumentation; ODI, Oswestry Disability Index; SS-PI, short-segment posterior instrumentation; VAS, visual analog scale

## Discussion

Historically, the initial management for OVF is primarily conservative, including short-term bed rest, analgesic medication, orthotic support, and the initiation of anti-osteoporotic medication to increase bone mineral density [10, 18]. Surgical management is generally reserved for patients who have failed conservative treatment. Nowadays, the clinical guidelines for managing OVF have been inconsistent, with no established consensus [19]. To address this inconsistency, the DGOU developed the OF classification and OF score. These tools are designed to assess OVF severity and guide treatment decisions. However, their reliability and validity have been evaluated in only a few studies, predominantly by the original developers. Notably, no prior research has comprehensively assessed the reliability of the OF score.

Our study demonstrated good interobserver and intraobserver reliability for the overall OF score, with ICCs of 0.774 and 0.833, respectively. These findings suggest strong agreement among raters and within the same rater over time. However, the reliability of individual OF score components varied. The OF classification achieved substantial agreement (kappa = 0.608), which was slightly lower than the kappa value reported by the DGOU (kappa = 0.63) [13]. Similar results were observed in recent studies, which reported kappa values of 0.59 [20] and ICC values of 0.62 [21].

The observed differences in reliability may stem from variations in evaluator experience. The DGOU study involved six expert spine surgeons, which likely contributed to higher agreement levels. Additionally, the use of advanced imaging techniques can impact reliability. At our institution, OVF patients are evaluated primarily via conventional radiography, with CT scans and MRIs employed selectively. This limited use of advanced imaging may hinder the accurate assessment of endplate involvement and posterior vertebral height, which is critical for distinguishing between the OF2, OF3, and OF4 classifications. Consequently, these challenges can affect the overall OF score evaluation in clinical settings, as changes in OF fracture type can lead to significant score alterations.

The “ongoing fracture” component of the OF score exhibited only moderate agreement, possibly due to difficulties in evaluating fracture progression with conventional radiography, especially in elderly patients with osteopenia and degenerative scoliosis. Conversely, score components such as “mobilization” and “health status” showed substantial agreement, whereas bone mineral density, pain measured by the VAS, and neurological deficits achieved near-perfect agreement. These latter components are more straightforward to assess, contributing to their higher reliability.

Despite adequate agreement levels, certain challenges remain in scoring specific components. There is a lack of clear definitions for sufficient analgesia and the precise site for bone mineral density measurement (e.g., total hip, femoral neck, or lumbar spine). Additionally, patient mobility varies widely, ranging from short-distance ambulation to the need for gait aids or caregiver assistance. These factors likely contribute to the lower kappa value observed in the mobilization component. Enhancing the reliability of the OF score may require more explicit definitions and standardized assessment protocols for these components.

The OF score provides therapeutic guidance with high accuracy (82.88%) and sensitivity (91.89%), demonstrating fair discriminative performance (AUC = 0.77). The ROC analysis identified a cutoff value of > 6.5, which aligns with the current threshold of an OF score ≥ 7 for recommending surgical treatment. However, the specificity of the OF score is relatively low (54.3%). Among patients with an OF score above 7, 16 patients (13.5%) were successfully treated conservatively. Notably, half of these patients (8/16) were classified as OF type 4 without neurological deficits or limited mobility. On follow-up X-rays, these patients showed no ongoing fractures, yet they reported pain with a VAS score ≥ 4. These findings suggest a risk of over-recommending surgical treatment when relying solely on the OF score. This could lead to unnecessary surgical interventions, particularly for type 4 OF patients, who may still be suitable candidates for conservative treatment despite an OF score exceeding 7 [22].

Our results are consistent with those of Ullrich et al. in the EOFTT study, which prospectively validated 518 OVF. They reported a high accuracy level (71%) but slightly lower discriminative ability (AUC = 0.684) for the OF score, with a cutoff value of 6.5 [14]. In contrast, our ROC analysis demonstrated a higher sensitivity (87.9% vs. 60%), indicating greater reliability in identifying OVF patients who genuinely need surgical intervention.

This study comprehensively evaluated three surgical treatments for OVF: stand-alone CA, SS-PI, and LS-PI. Regardless of the surgical strategy, all the treatments significantly improved the back pain VAS, ODI, EQ-VAS, and EQ-5D-5 L scores. LS-PI achieved the most substantial correction of the local kyphotic angle, whereas CA was associated with the greatest postoperative kyphotic progression after 1 year of follow-up. Additionally, the length of the construct (SS-PI vs. LS-PI) did not lead to significant differences in radiologic outcomes.

Most of our OVF patients were classified as having OF4 or OF3 injuries. According to the DGOU’s guidelines, LS-PI is recommended for OF4 injuries, and SS-PI is advised for OF3 injuries. However, nearly half of our patients (46%) were treated with stand-alone CA and achieved comparable clinical outcomes. Previous investigations have reported similar results. For instance, Ulrich et al. [22] evaluated OF4 in the EOFTT study and reported similar short-term outcomes across the three treatments. Gu et al. [23] also found comparable outcomes between CA and SS-PI but noted greater postoperative kyphotic progression and vertebral height loss in the CA group.

The kyphotic progression observed in the CA group may be attributed to alterations in load transfer and stress distribution caused by the cement, potentially leading to endplate damage or adjacent fractures [24, 25]. These cement-induced changes can result in postoperative kyphotic progression, especially in osteoporotic fractures with endplate injuries (OF4). However, our study found no significant difference in the rate of adjacent fractures between the treatment groups.

Despite the DGOU’s surgical recommendations, CA may be a viable treatment option for OVF, even in patients with loss of posterior wall integrity (OF3) or both types of endplate involvement (OF4). Nevertheless, the potential for postoperative kyphotic progression remains a major consideration, given that this condition can lead to late deformity and chronic back pain in OVF patients. These findings suggest that treatment decisions should not rely solely on the OF score but should also consider individual patient characteristics and clinical assessments.

### Limitations

Several limitations of this study must be acknowledged. First, the single-center, retrospective design may have led to an overestimation of the kappa values of certain components, such as bone mineral density, pain VAS score, and neurological deficits. In real-world settings, assessing bone mineral density in patients with acute OVF can be challenging, as these patients may be unable to tolerate supine positioning or experience prolonged waiting times. Alternative bone quality assessment methods, such as quantitative CT [26], could increase the accuracy and clinical utility of the OF score. Future studies should employ a multicenter, prospective design to strengthen generalizability and validity in real clinical practice. Second, the relatively low number of conservatively treated OVF patients, due to high rates of unavailable data and short follow-up periods, introduced the potential for selection bias. Consequently, the positive predictive value and negative predictive value of the OF score should be interpreted with caution. This limitation also restricts comparisons between conservative and surgical treatments. However, our primary aim was to evaluate the long-term outcomes of various surgical strategies recommended by the DGOU; thus, comparisons were limited to surgically treated patients.

## Conclusions

This study confirms the reliability and validity of the OF score, highlighting its utility in guiding treatment decisions for OVF, particularly in identifying patients suitable for conservative management. Stand-alone CA, SS-PI, and LS-PI are all effective surgical options for treating OVF. However, careful consideration is necessary when selecting CA for patients with OF3 or OF4 fractures because of the procedure’s limitations in correcting kyphotic angles and the risk of postoperative kyphotic progression.

## Data Availability

No datasets were generated or analysed during the current study.

## Abbreviations

AUC: Area under the curve
CA: Cement augmentation
CT: Computerized tomography
DGOU: German Society of Orthopedics and Trauma
ICC: Intraclass correlation coefficient
LS-PI: Long-segment posterior instrumentation
MRI: Magnetic resonance imaging
ODI: Oswestry Disability Index
OF: Osteoporotic fracture
OF score: Osteoporotic fracture treatment score
OVF: Osteoporotic vertebral compression fracture
ROC: Receiver operating characteristic
SS-PI: Short-segment posterior instrumentation
VAS: Visual analog scale
